# Effects of soft bracing or taping on a lateral ankle sprain: a non-randomised controlled trial evaluating recurrence rates and residual symptoms at one year

**DOI:** 10.1186/s13047-015-0069-6

**Published:** 2015-04-14

**Authors:** Ellen Kemler, Ingrid van de Port, Sandor Schmikli, Bionka Huisstede, Arno Hoes, Frank Backx

**Affiliations:** Department of Rehabilitation, Nursing Science and Sport, Rudolf Magnus Institute of Neuroscience, University Medical Centre Utrecht, Utrecht, PO Box 85500, 3508 GA The Netherlands; Consumer Safety Institute, Amsterdam, The Netherlands; Revant Rehabilitation Centre, Breda, The Netherlands; Julius Centre for Health Sciences and Primary Care, University Medical Centre Utrecht, Utrecht, The Netherlands

**Keywords:** Ankle sprains, Braces, Athletic tape, Recurrence

## Abstract

**Background:**

After sustaining an ankle sprain, taping is often the standard treatment in primary care. Ankle braces are sometimes used as an alternative. This study aimed to compare the effects of four weeks of soft bracing or taping following acute lateral ankle ligamentous sprain (ALALS) on sprain recurrence rates and residual symptoms at one year.

**Methods:**

In this pragmatic, non-randomised controlled trial, 157 adult participants with an ALALS caused by an inversion trauma were alternately allocated to a four week treatment with a soft brace (intervention group) or a four week treatment with ankle tape (control group) in order of presentation. The primary outcome was the 1-year incidence of the self-reported recurrence of ALALS. The secondary outcome was the occurrence of residual symptoms.

**Results:**

Baseline characteristics did not differ appreciably between the treatment groups. Thirteen patients (17%) in the brace group reported a re-injury versus 11 patients (14%) treated with tape, corresponding to a risk difference of 3.1% (relative risk 1.2, 95% CI 0.6 to 2.6). Cox regression analysis showed a hazard ratio of self-reported ankle sprain recurrence within 52 weeks in the brace group compared to the tape group of 0.9 (95% CI 0.4 to 1.9). After one year, patients in the brace group had poorer scores on the manual anterior ankle test, corresponding to a risk difference of 15.4% (RR 2.4, 95% CI 1.1 to 5.0).

**Conclusion:**

ALALS recurrences and residual symptoms appear to be similar at one year when an ALALS is treated with four weeks of soft bracing or taping.

**Trial registration:**

ISRCTN92030205

## Background

Ankle sprains are one of the most common musculoskeletal injuries. Reported incidence rates range from 2.2 sprained ankles per 1,000 person-years in the United States of America [1] to 5.3-7.0 sprained ankles per 1,000 person-years in Europe [2,3]. Ankle injury rates are high in both sports and the general population [4,5]. Of all the ankle injuries related to sports activities, 77% are ankle sprains [6]. An ankle sprain can cause pain and other symptoms, including residual symptoms such as muscle weakness, swelling, stiffness, and ankle instability. Residual symptoms are reported by 3 to 59% of the patients 3 to 24 months after the initial injury [7-12]. Recurrences of ankle sprain are also frequently reported [13].

Functional treatment including taping, bandaging and bracing of lateral ankle ligamentous sprains has been recommended instead of surgical repair and plaster cast or splint immobilisation since the early 1990s [14-17]. However, one study suggested that cast immobilisation might be more effective than functional treatment [18]. Several systematic reviews were unable to determine the most effective functional treatment strategy for acute lateral ankle ligamentous sprains (ALALS) [16,19]. ALALS are commonly treated with athletic ankle tape in primary care in the Netherlands. The guidelines of the Dutch College of General Practitioners for the treatment of ALALS recommends treatment consisting of immobilisation, compression, and elevation (ICE) during the first week, followed by ankle taping for six weeks [20].

Several disadvantages of treatment with ankle tape are known. Ankle tape cannot be applied when swelling and oedema are still clearly present. Skin care before treatment is needed, and even then, irritation of the skin by the tape is common. Finally, tape stability decreases approximately 14% after 30 minutes of exercise [21]. Compared to ankle tape, an ankle brace is easy to apply and to adjust by patients themselves. In addition, the risk of skin irritation is much lower, and ankle braces are reusable and washable. As an ankle brace seems to be more user friendly, it might also be more appropriate to treat ALALS. A soft brace is based on the principle of the functional tape bandage and it has been developed as an alternative to ankle tape treatment.

The purpose of this study was to compare the effects of four weeks of soft bracing or taping following ALALS on sprain recurrence rates and residual symptoms at one year. The incidences of ALALS recurrences and residual symptoms were expected to be similar in both treatment groups.

## Methods

This study was a pragmatic, non-randomised controlled trial.

### Participants and procedure

Patients aged 18 years and older and diagnosed with an ALALS caused by an inversion trauma were recruited from 20 family practices, nine physical therapy practices, the emergency department of a regional hospital and a university hospital located in the central part of the Netherlands. Patients were recruited between May 2006 and October 2008. Those aged younger than 18 years old were excluded from this study.

Patients with a possible ALALS were referred to the Department of Sports Medicine as soon as possible. The research assistant contacted the patients by phone and conducted the first screening for inclusion and exclusion criteria (age, multiple trauma, complicated trauma, history of surgery) by means of a short standard questionnaire. If the patient was eligible for inclusion in this study, the research assistant allocated them to the brace or tape group based on the order of presentation. The research assistant was blinded for the severity, see Appendix A of the inclusion injury, was not a medical expert, and was not responsible for determining the final eligibility of the patients. To check if the patients were indeed eligible for inclusion, a sports physician conducted a baseline assessment. The sports physician diagnosed an ALALS based on the following items: swelling of the injured ankle; any discoloration by haematoma; limited dorsiflexion in the injured ankle; clear tenderness at one or more anatomical locations related to the injured ankle; difference in the anterior drawer sign between the injured and contralateral ankle; and difference in the talar tilt test between the injured and contralateral ankle. Patients who had an ALALS caused by an eversion trauma, multiple trauma, or complicated trauma (including cartilage injuries, fractures and dislocation) or who had a history of ankle surgery were excluded. Patients diagnosed as having a mental illness or cognitive impairment were also excluded from this study. The medical ethics committee of the University Medical Centre Utrecht (UMCU) approved the protocol (protocol number 05/153). All participants gave written informed consent.

### Interventions

During the first five days after the ALALS, patients were treated with immobilisation, compression and elevation (ICE). As the diagnosis of an ALALS and inclusion were based on history and delayed physical examination at the UMC Utrecht [22,23], the allocated intervention started as soon as possible but at least within 14 days after the initial trauma. Only in case of very severe ankle swelling the ICE treatment was continued and the allocated intervention was postponed for a few additional days. At initial treatment, no specific pain medication protocol was prescribed.

### Intervention group

**Figure 1:**
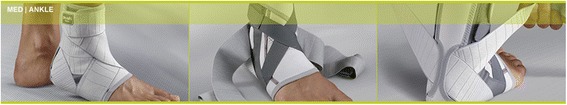
**Push Med ankle brace.**

The intervention group received instructions from the sports physician about using and applying the soft brace. The soft brace (Push® med Ankle Brace (Nea International bv)) [24] is based on the principles of the functional tape bandage (Figure 1). Participants were instructed to wear the soft brace for four weeks, except for at night and when taking a shower.

### Control group

The control group received usual care for ALALS for four weeks, which starts with ankle taping after ICE [20]. The general practitioner, primary care assistant, physical therapist, or plaster technician applied the athletic tape bandage. The health care professional decided on the most appropriate application technique for the specific patient. Patients were instructed to wear the ankle tape for four weeks. In the Netherlands, it is common to wear a tape bandage for two weeks before refreshing [20]. Two weeks after the first application the tape was refreshed by the healthcare professional. When necessary, due to the loss of stability or hygiene, the tape bandage in the control group was replaced earlier, by the same healthcare professional. After four weeks the ankle tape was removed and discontinued.

### Data collection and outcomes

Baseline data were obtained by the sports physicians and consisted of a clinical history and a physical examination of the ankle. All the sports physicians received standardised training for measuring outcomes. All participants were invited for a reassessment by one of the sports physicians one year after treatment allocation. This assessment included the same physical examination as at the baseline. In addition, participants were asked about re-injuries, current residual symptoms and pain, and the use of additional therapy or aids. The sports physicians could not be blinded to treatment allocation.

The primary outcome was the proportion of participants with a recurrence of ALALS within one year. A recurrence of ALALS was defined as a new inversion trauma of the same ankle, reported by the patient during a year following treatment allocation. To register the recurrences of ALALS participants filled in online questionnaires 5, 9, 13, 26, and 39 weeks after the initial injury. Participants had to answer the following questions: “Did you re-injure your ankle after the start of this study?” (answer: yes or no) and “What was the nature of the injury (response options: sprain, broken ankle, overuse injury, I don’t know, other). Ankle fractures or overload of the affected ankle were not regarded as recurrences of ALALS.

The secondary outcome was the occurrence of residual symptoms. This included: (i) residual swelling (substantial/moderate/minimal/no), (ii) functional outcome (no limited dorsiflexion/injured ankle better than non-injured ankle/limited dorsiflexion), and (iii) passive and active stability assessed by the sports physician based on clinical interpretation. Passive stability was defined as ligament stability. The manual anterior ankle test as described by Van Dijk et al. [25] and the talar tilt test were used to measure ligament stability. Active stability was defined as muscular stability while performing a one-leg stance test – four variations of the one-leg stance test with an increasing difficulty were used to measure this type of stability [26]. The four variations were: (i) eyes open, (ii) eyes closed, (iii) eyes closed and knee in 45 degrees flexion, (iv) eyes closed, knee in 45 degrees flexion and standing on forefoot [26]. Both legs were tested barefoot. The time in seconds a participant could stand on one leg was measured. The score was then dichotomised with a ‘successful’ test recorded if the participant could perform the one-leg stance test for 15 seconds or more. Standing on one leg for less than 15 seconds meant that a patient had failed the one-leg stance test.

Pain in the ankle joint during walking, running, pivoting and jumping was reported by the patient. Participants were classified as having pain (yes/no) when they reported pain in the ankle joint during at least one of these activities. Although no specific pain medication protocol was prescribed in this study, participants were asked whether they did use pain medication or not. This was similar for manual therapy and physiotherapy.

Self-reported data on the compliance of wearing the soft brace or tape were collected once in the five-week online questionnaire.

### Sample size

The incidences of ALALS recurrences were expected to be similar in both treatment groups. However, a clinically worthwhile difference for interventions, (i.e. the difference or the ratio of the cumulative incidence of re-injury between the two treatments) was not available to use for an *a priori* sample size calculation. Thus, we aimed to include as many participants as possible in this study within a period of 30 months.

### Statistical analyses

Baseline characteristics were described and compared using chi-square tests and independent sample *t*-test. The one year cumulative incidence of self-reported recurrence of ALALS and the prevalence of residual symptoms at one year for both treatment groups, and risk differences and relative risks (RR) with 95% confidence intervals were calculated using crosstabs with risk estimation. In addition, when comparing the risk for re-injury, Cox regression analysis (hazard ratio: HR) was also used, taking into account the time between initial injury and re-injury. In the analyses of self-reported recurrence of ALALS, all participants were included as specified by the allocation to the treatment groups (i.e. for this analysis, we used the intention to treat principle). In the analyses of residual symptoms all participants who were reassessed by a sports physician one year after the treatment allocation were included as specified by the allocation to the treatment groups. Participants who underwent no physical examination after one year of follow-up were excluded from the analyses of residual symptoms. For compliance, the RR was calculated using crosstabs with risk estimates. In case of baseline differences between the two groups, we planned multivariable regression analyses (logistic regression for the one year incidence and Cox regression) to adjust for potential confounders.

Data for all the respondents were analysed using the IBM SPSS statistical software package, version 18.0.

## Results

**Figure 2:**
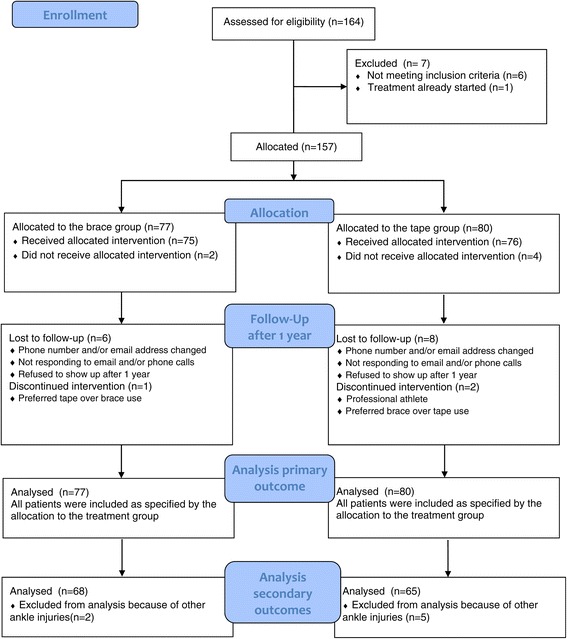
**Trial profile.**

A total of 164 patients were assessed for eligibility in this study (Figure 2). Seven patients were excluded due to the following reasons: age under 18 years (n = 3), ankle fracture (n = 2), treatment already started before treatment allocation (n = 1) and an eversion ankle sprain (n = 1). The age of the 157 included participants ranged from 18 to 64 years (mean 31.1 years); 88 participants (56%) were male. One hundred (64%) of the reported ALALS occurred during sports participation. On average, the allocated treatment started 6.1 days (SD 2.3) and 5.8 days (SD 2.0) after the initial injury in the brace and tape group, respectively. Baseline characteristics did not differ appreciably between the treatment groups (Table 1).

**Table 1:** **Comparison of demographic and baseline characteristics between groups**

	**Soft bracing N = 77**	**Taping N = 80**	***P-*** **value**
Mean age in years (SD)	30.7 (11.3)	31.4 (12.0)	*0.708*
Time from injury to start of treatment, mean in days (SD)	6.1 (2.3)	5.8 (2.0)	*0.467*
Gender, male, n (%)	41 (53.2)	47 (58.8)	*0.487*
Sports participants^1^, n (%)	66 (85.7)	66 (83.3)	*0.582*
Injury severity^2^			*0.110*
- Mild, n (%)	28 (36.4)	17 (21.3)	
- Moderate, n (%)	38 (49.4)	48 (60.0)	
- Severe, n (%)	11 (14.3)	15 (18.8)	
Previous sprains of the injured ankle			*0.178*
- Yes, n (%)	34 (44.2)	24 (30.0)	
- No, n (%)	32 (41.6)	43 (53.8)	
- Unknown, n (%)	11 (14.3)	13 (16.3)	

*SD, Standard Deviation.*

^*1*^ *Participating in sports in the week prior to the occurrence of the initial injury.*

^*2*^ *For a detailed explanation of Injury severity, see* *Appendix A* *.*

### Additional therapy and the use of medication

A total of 57 participants (36%) visited a physical or manual therapist during the treatment period and one year follow-up after the initial trauma; of these, 30 were in the brace group (mean of 8 visits per participant) and 27 in the tape group (mean of 11 visits per participant). Thirty seven participants (24%) used some kind of medication; of these, 23 were in the brace group and 14 were in the tape group. No significant differences were found for the use of medication and additional therapy.

### Re-injuries

Within 52 weeks after the initial trauma, 13 of the 77 participants (17%) in the brace group compared to 11 of the 80 participants (14%) in the tape group reported a re-injury, corresponding to a risk difference of 3.1% (RR 1.2, 95% CI 0.6 to 2.6). Two participants (3%) in the brace group and no participants in the tape group reported a re-injury of the affected ankle within the treatment period of four weeks. Cox regression analysis showed a HR for self-reported ankle sprain recurrence within 52 weeks in the brace group compared to the tape group of 0.9 (95% CI 0.4 to 1.9).

### Residual symptoms

Seventeen participants (11% of the total sample) were lost to follow-up and therefore did not have a physical examination after one year. Another 7 participants (4% of the total sample) were excluded from the analyses because of other ankle injuries (in total 9 and 15 respondents in the brace and tape group, respectively). The 24 participants (15% of the total sample) who were excluded from the residual symptoms analyses did not have different baseline characteristics to those included in the analyses.

One year after the initial ankle trauma, no differences between the groups were reported on swelling, functional outcome, active stability and pain (Table 2). However, participants in the brace group showed poorer scores on the manual anterior ankle test, corresponding to a risk difference of 15.4% (RR 2.4, 95% CI 1.1 to 5.0).

**Table 2:** **Residual symptoms in ALALS participants after one year of follow-up**

	**Soft bracing N = 68** ^**1**^		**Taping N = 65** ^**1**^		**RR (95%CI)**	***P*** **-** ***value***
Swelling, n (%)	11	(16.2)	12	(18.5)	0.9 (0.4 to 1.8)	*0.820*
Functional outcome (limited flexion), n (%)	20	(29.4)	18	(27.7)	1.1 (0.6 to 1.8)	*0.850*
Passive instability (anterior ankle test), n (%)	20	(29.4)	8	(12.3)	2.4 (1.1 to 5.0)	*0.019*
Passive instability (talar tilt test), n (%)	16	(23.9)	17	(26.2)	0.9 (0.5 to 1.6)	*0.842*
Active instability of injured ankle, n (%)						
Active instability with, n (%):						
- one leg stance, eyes open	5	(7.4)	4	(6.2)	1.2 (0.3 to 4.3)	*1.000*
- one leg stance, eyes closed	48	(70.6)	39	(60.0)	1.2 (0.9 to 1.5)	*0.208*
- one leg stance, eyes closed, with knee in 45° flexion	50	(73.5)	44	(67.7)	1.1 (0.9 to 1.4)	*0.568*
- one leg stance, eyes closed, with knee in 45° flexion, standing on the forefoot	68	(100.0)	65	(100.0)	1.0 (1.0 to 1.0)	*1.000*
Pain in the ankle joint during walking, running, pivoting and jumping, n (%)	21	(27.7)	18	(30.9)	1.1 (0.7 to 1.9)	*0.707*

^*1*^ *17 participants underwent no physical exam after one year of follow-up and 7 participants were excluded from the analyses because of other ankle injuries (tape n = 15; brace n = 9).*

### Compliance

A total of 81 participants (54%) completed their prescribed four weeks treatment as instructed; of those, 38 were in the brace group and 43 in the tape group. No difference in compliance between the treatment groups was found (RR 1.1, 95% CI 0.8 to 1.6). The main reason for not completing the four week treatment as instructed was skin irritation (39% in total; 27% in the brace group and 52% in the tape group).

## Discussion

In our study comparing four-week treatment with a soft brace and ankle tape in participants with ALALS, the one-year incidence of re-injury was comparable in both groups with a risk difference of 3.1% (RR 1.2, 95% CI 0.6 to 2.8). The proportion of participants with re-injuries found in this study (15%) is similar to other studies [13].

For passive stability (ligament stability), using the manual anterior ankle test, a difference between the two treatment groups was found at 52 weeks in favour of the tape group. Passive stability did not differ at baseline, but the test was often performed a couple of days after the initial ankle injury. Thus, it is unknown whether the difference in passive stability already existed before the start of this trial (and the initial ankle injury) or was related to the allocated treatment. In this study, the manual anterior ankle test and talar tilt test were used. The use of these manual tests is difficult to discuss because of their subjective nature, and the inability to produce reproducible and quantitative results [27]. Both manual tests are, however, still frequently used to estimate ankle joint laxity. Several devices have been developed to objectively measure ankle joint laxity; for example, the dynamic anterior ankle tester (DAAT) [28]. At the start of our study, the reliability of this device had been found to be high, but its validity needed further investigation [29]. Furthermore, at the time of the study, the apparatus itself was not suitable for clinical practice [28]. As the manual anterior ankle test and talar tilt test were frequently used in clinical practice, we chose to use these manual tests in this pragmatic trial.

Active stability, or muscular stability, did not differ after one year. Several earlier studies have demonstrated that passive or mechanical stability is related to active stability or functional stability [30,31]. However, a recent study demonstrated the opposite [32]. In 2002, Hertel developed a model of functional and mechanical insufficiency, which can be helpful in explaining the causative spectrum related to Chronic Ankle Instability (CAI) [33]. In this model two subgroups are included, classified according to the presence of either mechanical instability or functional instability. When both mechanical and functional instability are present in a patient, a third subgroup of recurrent ankle sprains arises. In a recent study, Hiller et al. (2011) proposed a modification of the Hertel model for CAI. In the new model mechanical instability, perceived or functional instability, and recurrent ankle sprain can exist independently or in combination, with seven subgroups of patients now differentiated [34]. The results of our trial seem to fit this latest model proposed by Hiller et al. However, it was not our purpose to evaluate the relationship between functional and mechanical ankle instability.

In the early 1990s, Twellaar et al. compared one of the first prototypes of the soft brace with ankle tape [35]. After an average follow-up period of 2.3 years, tenderness at the lateral ligaments occurred more often after applying tape. They concluded that soft bracing might be preferred over ankle taping for practical reasons, with a lower risk for skin irritation being one of these. The results of our study showed that skin irritation was the main reason for not completing the prescribed treatment, especially in the tape group. The percentage of participants who were not compliant to the treatment was almost twice as high in the tape group compared to the brace group (52% to 27%).

Several studies have compared ankle braces with elastic wrapping or Tubigrip™ [18,36,37]. In a recent review, Kemler et al. compared the effectiveness of ankle brace treatment with other functional treatment and classified the evidence for the effectiveness of ankle bracing on functional outcomes as strong [16]. This result was based on studies from Boyce et al. [37], Beynnon et al. [36], Lamb et al. [18], Karlsson et al. [38] and Leanderson and Wredmark [39]. The results of this review seem to contrast with our findings. However, the heterogeneity of these studies should be taken into account. For example, the studies had different follow-up periods, used other types of ankle braces, the ankle brace was compared with treatment methods other than tape, and different outcome measurements were used.

Several limitations of our pragmatic trial should be discussed. Firstly, different application techniques for ankle taping were used by the clinicians involved. The use of different application methods was, however, in line with usual care as described in the guideline of the Dutch College of General Practitioners. In all cases, the tape was applied by a health care professional using the most appropriate method for that patient. Secondly, for logistical reasons, the allocation of participants to the treatment groups was based on the order of presentation, and thus not on a formal randomisation scheme. The comparability of the two groups (Table 1) clearly indicates that our allocation scheme resulted in groups with similar participant characteristics and therefore, similar prognosis. Thirdly, in this study an ‘all complaints’ definition of recurrent ankle sprains was used instead of a ‘medical attention’ or ‘time-loss definition’ [40]. The data may be a good representation of the total burden of ALALS, but their validity may be suspect. Participants were required to judge and decide for themselves whether their new sprain was a re-injury according to the following definition: ‘a re-injury is an ankle sprain (injury) to the same ankle’. As a re-injury was not assessed by a medical professional, no detailed information about the severity of these new injuries was available. Fourthly, an *a priori* sample size calculation was not performed. A post-hoc power analysis was not performed either because it is controversial regarding its risk of a “power approach paradox”, whereby if a significant difference is not found and a post hoc power analysis is performed after the study, the study will automatically be found to be underpowered. However, it is possible that there is truly no significant difference between the two treatments (i.e. that both treatments are in fact equal to each other). Indeed, our findings indicate that the effects of both treatments are comparable. Although, the 95% confidence intervals are rather wide, so we cannot rule out that our study may have been underpowered and not able to identify smaller differences in treatment effects. Finally, the assessors in our study, the sports physicians, were not blinded to group assignment. The sports physicians had to instruct all participants about the allocated treatment method and had to apply the ankle brace if a participant was assigned to the brace group. We do not expect that this has influenced our results, but we cannot discount the possibility of ascertainment bias. The seven sports physicians who participated in this study were all independent and had no conflicts of interest. Furthermore, the primary outcome was self-reported by the participants.

Our results underline the considerations that both an ankle brace and ankle tape can be used in the treatment of ALALS in primary care. According to Kerkhoffs et al. [41], a lace-up brace or a semi-rigid brace is preferable and recommended in the treatment of ALALS. However, in (professional) sports, the use of tape can also be considered [41]. Van den Bekerom et al. [42] also concluded that either tape, a semi-rigid ankle brace, or a lace-up brace can be used in functional treatment of ALALS.

Functional treatment (including tape, bandage or brace) of ALALS has been recommended instead of surgical repair and plaster cast or splint immobilisation since the early 1990s [14-17]. However, the discussion regarding the use of a short period of immobilisation, followed by functional treatment, exists again [18,41-43]. Additional research focusing on effectiveness of the treatment of ALALS needs to be conducted. In addition, while both the ankle brace and ankle tape can be used for ALALS treatment, an economic evaluation including treatment costs, medical costs and costs due to absence from paid work, unpaid work and school would provide more insight into the cost-related aspects of both treatments.

## Conclusions

The results after one-year follow-up indicate that in participants with ALALS, treatment with a soft brace or ankle tape shows similar effects on the incidence of ALALS recurrence and on residual symptoms.

## Appendix A

The injury severity score (range 0-6) was determined by the sports physician at baseline based on six items, scored as 1 when present and 0 when absent:

1. swelling of the injured ankle;
2. any discoloration by heamatoma;
3. limited dorsiflexion in the injured ankle;
4. clear tenderness at one or more anatomical locations related to the injured ankle;
5. difference in the anterior drawer sign between the injured and contralateral ankle, and;
6. difference in the talar tilt test between the injured and contralateral ankle.

Injury severity was based on the total score (0-6) in relation to the number of days between the onset of the initial trauma and assessment of the injury severity score, and was categorised as follows:

- Severe lateral ankle ligamentous sprain (score of 6 and physical exam at least four days after the injury).
- Moderate lateral ankle ligamentous sprain (score of 6 and physical exam on days one-three after the injury *OR* score of 5 or 4 and physical exam at least four days after the injury).
- Mild lateral ankle ligamentous sprain (all other combinations).

## References

[1] Waterman BR, Owens BD, Davey S, Zaccili MA, Belmont PJ (2010). The epidemiology of ankle sprains in the United States. J Bone Joint Surg Am.

[2] Bridgman SA, Clement D, Downing A, Walley G, Phair I, Maffulli N (2003). Population based epidemiology of ankle sprains attending accident and emergency units in the West Midlands of England, and a survey of UK practice for severe ankle sprains. Emerg Med J.

[3] Hølmer P, Søndergaard L, Konradsen L, Nielsen PT, Jørgensen LN (1994). Epidemiology of sprains in the lateral ankle and foot. Foot Ankle Int.

[4] Kemler E, Port van de I, Valkenberg H, Hoes AW, Backx FJG: Ankle injuries in the Netherlands: Trends over 10–25 years. Scand J MedSci Sports. doi:10.1111/sms.12248. 10.1111/sms.12248 24840653

[5] Schmikli SL, Backx FJG, Kemler HJ, Van Mechelen W (2009). National survey on sports injuries in the Netherlands: target populations for injury prevention programs. Clin J Sport Med.

[6] Fong DT, Hong Y, Chan LK, Yung PS, Chan KM (2007). A systematic review on ankle injury and ankle sprain in sports. Sports Med.

[7] Cetti R (1982). Conservative treatment of injury to the fibular ligament of the ankle. Br J Sports Med.

[8] Gerber JP, Williams GN, Scoville CR, Arciero RA, Taylor DC (1998). Persistent disability associated with ankle sprains: a prospective examination of an athletic population. Foot Ankle Int.

[9] Handoll HHG, Rowe BH, Quinn KM, de Bie R (2001). Interventions for preventing ankle ligament injuries. Cochrane Database Syst Rev.

[10] Hubbard TJ, Hicks-Little CA (2008). Ankle ligament healing after an acute ankle sprain: an evidence-based approach. J Athl Train.

[11] Yeung MS, Chan KM, So CH, Yuan WY (1994). An epidemiological survey on ankle sprain. Br J Sports Med.

[12] Zwipp Z, Hoffmann R, Thermann H, Wippermann BW (1991). Rupture of the ankle ligaments. Int Orthop.

[13] van RM R, van Os AG, Bernsen RM, Luijsterburg PA, Koes BW, Bierma-Zeinstra SM (2008). What is the clinical course of acute ankle sprains? A systematic literature review. Am J Med.

[14] Jones MH, Amendola AS (2007). Acute treatment of inversion ankle sprains: immobilization versus functional treatment. Clin Orthop Relat Res.

[15] Kannus P, Renström P (1991). Treatment for acute tears of the lateral ligaments of the ankle: operation, cast, or early controlled mobilization. J Bone Joint Surg Am.

[16] Kemler E, van de Port I, Backx F, van Dijk CN (2011). A systematic review on the treatment of acute ankle sprain Braces versus other functional treatment types. Sports Med.

[17] Kerkhoffs GMMJ, Rowe BH, Assendelft WJJ, Kelly K, Struijs PA, van Dijk CN (2001). Immobilisation for acute ankle sprain: a systematic review. Arch Orthop Trauma Surg.

[18] Lamb SE, Marsh JL, Hutton JL, Nakash R, Cooke MW (2009). Collaborative Ankle Support Trial (CASTGroup): Mechanical supports for acute severe ankle sprain: a pragmatic, multicentre, randomized controlled trial. Lancet.

[19] Kerkhoffs GM, Struijs PA, Marti RK, Assendelft WJ, Blankevoort L, van Dijk CN (2002). Different functional treatment strategies for acute lateral ankle ligament injuries in adults. Cochrane Database Syst Rev.

[20] NHG (Dutch General Practitioner College): Guideline on ankle sprain (2000) [online; in Dutch]. Available from URL: https://www.nhg.org/standaarden/samenvatting/enkelbandletsel (guideline was revised in 2012)

[21] Alt W, Lohrer H, Gollhofer A (1999). Functional properties of adhesive ankle taping: neuromuscular and mechanical effects before and after exercise. Foot Ankle Int.

[22] van Dijk CN, Lim LS, Bossuyt PM, Marti RK (1996). Physical examination is sufficient for the diagnosis of sprained ankles. J Bone Joint Surg Br.

[23] van den Bekerom MPJ, Struijs PAA, Blankevoort L, Welling L, van Dijk CN, Kerkhoffs GM (2012). What is the evidence for RICE therapy in the treatment of ankle sprains? Systematic review of literature. J Athl Train.

[24] De Clercq DL (1997). Ankle bracing in running: the effect of a Push type medium ankle brace upon movements of the foot and ankle during the stance phase. Int J Sports Med.

[25] van Dijk CN, Mol BW, Lim LS, Marti RK, Bossuyt PM (1996). Diagnosis of ligament rupture of the ankle joint. Physical examination, arthrography, stress radiography and sonography compared in 160 patients after inversion trauma. Acta Orthop Scand.

[26] Trojian TH, McKeag DB (2006). Single leg balance test to identify risk of ankle sprains. Br J Sports Med.

[27] Fujii T, Luo Z-P, Kitoaka HB, An KN (2000). The manual stress test may not be sufficient to differentiate ankle ligament injuries. Clin Biomech (Bristol, Avon).

[28] Kerkhoffs GM, Blankevoort L, van Dijk CN (2005). A measurement device for anterior laxity of the ankle joint complex. Clin Biomech (Bristol, Avon).

[29] Kerkhoffs GM, Blankevoort L, Sierevelt IN, Corvelein R, Janssen GH, van Dijk CN (2005). Two ankle joint laxity testers: reliability and validity. Knee Surg Sports Traumatol Arthrosc.

[30] Karlsson J, Andreasson GO (1992). The effect of external ankle support in chronic lateral ankle joint instability: an electromyographic study. Am J Sports Med.

[31] Tropp H, Odenrick P, Gillquist J (1985). Stabilometry recordings in functional and mechanical instability of the ankle joint. Int J Sports Med.

[32] Hirai D, Docherty CL, Schrader J (2009). Severity of functional and mechanical ankle instability in an active population. Foot Ankle Int.

[33] Hertel J (2002). Functional Anatomy, Pathomechanics, and Pathophysiology of Lateral Ankle Instability. J Athl Train.

[34] Hiller CE, Kilbreath SL, Refshauge KM (2011). Chronic ankle instability: evolution of the model. J Athl Train.

[35] Twellaar M, Veldhuizen JW, Verstappen FT (1993). Ankle sprains: comparison of long-term results of functional treatment methods with adhesive tape and bandage (“brace”) and stability measurement. Unfallchirurg.

[36] Beynnon BD, Renström PA, Haugh L, Uh BS, Barker H (2006). A prospective, randomized clinical investigation of the treatment of first time ankle sprains. Am J Sports Med.

[37] Boyce SH, Quigley MA, Campbell S (2005). Management of ankle sprains: a randomised controlled trial of the treatment of inversion injuries using an elastic support bandage or an Aircast ankle brace. Br J Sports Med.

[38] Karlsson J, Eriksson BI, Swärd L (1996). Early functional treatment for acute ligament injuries of the ankle joint. Scand J Med Sci Sports.

[39] Leanderson J, Wredmark T (1995). Treatment of acute ankle sprain. Comparison of a semi-rigid ankle brace and compression bandage in 73 patients. Acta Orthop Scand.

[40] Clarsen B, Bahr R (2014). Matching the choice of injury/illness definition to study setting, purpose and design: one size does not fit all!. Br J Sports Med.

[41] Kerkhoffs GM, van den Bekerom M, Elders LA, van Beek PA, Hullegie WA, Bloemers GM (2012). Diagnosis, treatment and prevention of ankle sprains: an evidence-based clinical guideline. Br J Sports Med.

[42] van den Bekerom MP, Kerkhoffs GM, McCollum GA, Calder JD, van Dijk CN (2013). Management of acute lateral ankle ligament injury in the athlete. Knee Surg Sports Traumatol Arthrosc.

[43] Petersen W, Rembitzki IV, Koppenburg AG, Ellermann A, Liebau C, Brüggemann GP (2013). Treatment of acute ankle ligament injuries: a systematic review. Arch Orthop Trauma Surg.

